# Analysis of predictive factors of unforeseen nodal metastases in resected clinical stage I NSCLC

**DOI:** 10.3389/fonc.2023.1229939

**Published:** 2023-11-07

**Authors:** Filippo Tommaso Gallina, Daniele Marinelli, Riccardo Tajè, Daniele Forcella, Gabriele Alessandrini, Fabiana Letizia Cecere, Francesca Fusco, Paolo Visca, Isabella Sperduti, Vincenzo Ambrogi, Federico Cappuzzo, Enrico Melis, Francesco Facciolo

**Affiliations:** ^1^ Thoracic Surgery Unit, IRCCS Regina Elena National Cancer Institute, Rome, Italy; ^2^ Department of Experimental Medicine, Sapienza University, Rome, Italy; ^3^ Medical Oncology 2, IRCCS Regina Elena National Cancer Institute, Rome, Italy; ^4^ Department of Pathology, IRCCS Regina Elena National Cancer Institute, Rome, Italy; ^5^ Biostatistics, IRCCS Regina Elena National Cancer Institute, Rome, Italy; ^6^ Department of Thoracic Surgery, Tor Vergata Policlinic, Rome, Italy

**Keywords:** early stage NSCLC, upstaging, stage I, nodal disease, lymphadenectomy

## Abstract

**Background:**

Despite notable advances made in preoperative staging, unexpected nodal metastases after surgery are still significantly detected. In this study we aim to analyze the upstaging rate in patients with clinical stage I NSCLC without evidence of nodal disease in the preoperative staging who underwent lobectomy and radical lymphadenectomy.

**Methods:**

Patients who underwent lobectomy and systematic lymphadenectomy for clinical stage I NSCLC were evaluated. Exclusion criteria included the neoadjuvant treatment, incomplete resection and no adherence to preoperative guidelines.

**Results:**

A total of 297 patients were included in the study. 159 patients were female, and the median age was 68 (61 - 73). The variables that showed a significant correlation with the upstaging rate at the univariate analysis were the number of resected lymph nodes and micropapillar/solid adenocar-cinoma subtype. This result was confirmed in the multivariate analysis with a OR= 2.545 (95%CI 1.136-5.701; p=0.02) for the number of resected lymph nodes and a OR=2.717 (95%CI 1.256-5.875; p=0.01) for the high-grade pattern of adenocarcinoma.

**Conclusion:**

Our results showed that in a homogeneous cohort of patients with clinical stage I NSCLC, the number of resected lymph nodes and the histological subtype of adenocarcinoma can significantly be associated with nodal metastasis.

## 1 Introduction

Non-Small Cell Lung Cancer (NSCLC) is the most frequent lung cancer subtype and represents the leading cause of cancer-related deaths in the world. Stage I NSCLC has an estimated 5-year overall survival rate of 90% after standard-of-care radical surgical resection. Metastatic spread to locoregional lymph nodes has a detrimental prognostic effect; standard-of-care (neo)adjuvant chemotherapy in node-positive NSCLC leads to improved disease-free survival and overall survival (1, 2). Thus, hilum-mediastinal staging for early detection of nodal metastases has a pivotal role in the multidisciplinary management of early-stage NSCLC. Preoperative clinical and invasive mediastinal staging hold the promise to accurately detect nodal metastases (3). Moreover, the survival benefit associated with lymphadenectomy has led to inconclusive results and questioned the degree to which hilar and mediastinal lymph nodes should be harvested (4). As a result, 42.4% of patients have no lymph nodes harvested at pulmonary resection for lung cancer and base their nodal staging solely on preoperative clinical or invasive staging (5). Despite the accuracy of preoperative staging procedures in detecting metastatic lymph nodes, a high rate of postoperative pathological upstaging is still detected (6). Indeed, recent reports have shown upstaging rates up to 25% in patients with early-stage NSCLC at surgery (7). Failure to identify these unexpected nodal metastases may lead to undertreatment. Furthermore, while neoadjuvant chemoimmunotherapy was recently shown to lead to clinically meaningful and statistically significant improvements in disease-free survival in stage I-III NSCLC over neoadjuvant chemotherapy, stage II-III tumors are likely to benefit most from this treatment option; baseline nodal staging is therefore critical to accurately advise multimodal treatment. By analyzing the surgical and histological features in a population of patients with clinical stage I NSCLC who underwent lobectomy and systematic lymphadenectomy, we aimed to identify the key factors contributing to upstaging in this particular cohort.

## 2 Materials and methods

The study was designed as a single-center, retrospective analysis of patients with clinical stage I NSCLC who underwent lobectomy and radical lymphadenectomy.

### 2.1 Inclusion criteria

- Patients with clinical stage I lung adenocarcinoma (LUAD).
- Complete preoperative staging in accordance with guidelines.
- Lobectomy and systematic hilum-mediastinal lymphadenectomy (in accordance with the IASLC definition of complete lymph node dissection of both N1 and N2 stations) with robotic surgery.

### 2.2 Exclusion criteria

- Patients with clinical stage II-III-IV.
- Sublobar resections and wedge resections.
- Incomplete lymphadenectomy.
- Patients who underwent preoperative chemotherapy or radiotherapy.

The aim of this study was to evaluate the association between surgical and histological variables with upstaging in patients with clinical stage I NSCLC who underwent radical surgical resection; all included patients received robotic surgery according to the preferences of the institution.

### 2.3 Preoperative staging

Prior to the surgical interventions, comprehensive preoperative investigations were conducted, including brain, thoracic, and upper abdominal computed tomography (CT) scans, as well as F18-fluorodeoxyglucose positron emission tomography (FDG-PET). These investigations were crucial to determine the absence of multiple pulmonary lesions and to rule out the presence of hepatic, adrenal, or brain metastases. Additionally, the status of hilar and mediastinal lymph nodes was assessed using these imaging modalities. In cases where indicated, a brain MRI was performed to ensure the exclusion of brain metastasis. To further evaluate the lymph node status, lymph nodes larger than 1 cm along the shortest axis or PET-CT avid nodes with standardized uptake value >1.5 underwent endoscopic or endobronchial ultrasonography fine-needle biopsy to exclude metastatic involvement. If clinically warranted, bone scintigraphy was also conducted. All patients provided informed consent for lobectomy before undergoing the surgical procedures.

### 2.4 Surgical technique

In all patients, a radical hilum mediastinal lymphadenectomy was performed following the guidelines. For tumors on the right side, systematic exploration of the paratracheal stations (2R and 4R), subcarinal station (7), paraoesophageal station (8), and inferior pulmonary ligament station (9) was carried out. For tumors on the left side, lymphadenectomy of the aorto-pulmonary window (5-6), subcarinal stations (7), para-oesophageal station (8), and inferior pulmonary ligament station (9) was usually performed. At the conclusion of the procedure, one chest tube was typically inserted using the camera port.

After surgery, all formalin-fixed paraffin-embedded (FFPE) tissue sections were reviewed by pathologists for histopathological confirmation and tumor content assessment. Predominant invasive LUAD histologic subtypes were classified as lepidic, acinar, papillary, micropapillary, or solid.

The primary endpoint was upstaging at surgical resection. Statistical analysis was performed by an experienced bio-statistician using SPSS 20 (IBM SPSS Statistics, IBM Corporation, Chicago, IL). Descriptive statistics were calculated and expressed as median and interquartile ranges. Groups were compared using t-tests for continuous variables and chi-square for categorical data. A multivariable logistic regression model with stepwise regression was used (with forward selection, enter limit, and remove limit set at p = 0.10 and p = 0.15, respectively) to identify independent factors associated with the primary outcome measure. Cut-off for linear variables were calculated with ROC curve analysis.

## 3 Results

**Figure 1:**
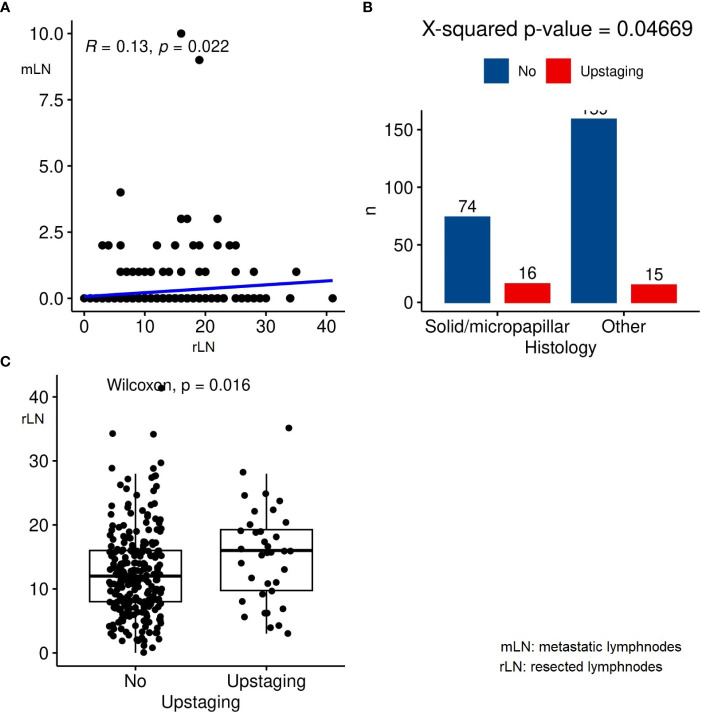
**(A)** Linear correlation between the resected lymph nodes and metastatic lymph nodes; **(B)** Correlation between adenocarcinoma subtypes and upstaging; **(C)** Correlation between resected lymph nodes and upstaging.

From January 2016 to September 2022, a total of 713 lobectomies and radical lymphadenectomies were performed for early stage NSCLC, of which 297 were clinical stage I. Of these, 159 patients were male. The clinical and pathological characteristics are reported in **Table 1**. The median age at diagnosis was 68 years (range: 61-73 years). A history of smoking was reported in 169 patients (56.9%). The median tumor diameter was 18 mm (range: 7-28 mm). A history of other cancers 5 years before the lung cancer diagnosis was reported in 15.8% of patients. All operations were carried out using robotic technology. Surgery was performed numerically more frequently on the right side (57.6%). According to the guidelines, all patients underwent the removal of at least 4 nodal stations. The median number of resected nodes was 12 (range: 8-16). The majority of patients presented a single metastatic nodal station, and the median number of metastatic lymph nodes after surgery was 2 (range: 1-4). After surgery, the majority of cases were adenocarcinoma, and one-third of patients showed a solid or micropapillary subtype (30.6%). In general, 36 (12.1%) patients reported upstaging, of whom 15 patients had mediastinal upstaging. As shown in **Figure 1A**, there was a significant but weak correlation between the number of resected and metastatic nodes in the adenocarcinoma group (p=0.022, ρ=0.13) upstaging was more frequent among micropapillary and solid adenocarcinoma subtypes (χ2 test, p= 0.04669, **Figure 1B**). Tumors with upstaging at surgical resection had a higher number of resected nodes (Wilcoxon p=0.016, **Figure 1C**).

**Table 1:** Surgical and histological characteristic of the total population and the upstaging and nonupstaging group.

	Total population (297)	Non upstaging (261)	Upstaging (36)	p- value
Age (median, IQR)	68 (61 – 73)	68 (60 – 73)	67 (61.75 – 73.25)	0.1
Sex (female), n (%)	138 (46.5)	125 (47.9)	13 (36.1)	0.4
Actual smokers, n (%)	169 (56.9)	146 (55,9)	23 (63.9)	0.3
Previous cancers, n (%)	47 (15.8)	42 (16.1)	5 (13.9)	0.3
Side (right), n (%)	171 (57.6)	165 (63.2)	15 (41,7)	0.1
Tumor diameter, (median, IQR)	18 (7-28)	17 (8-28)	18 (6-29)	0.7
Squamous histology, n (%)	31 (10.4)	26 (10)	5 (13.9)	0.2
Micropapillary or solid adc, n(%)	91 (30.6)	75 (28.7)	16 (44.4)	<0.05
Number of resected nodes (median, IQR)	12 (8–16)	12 (8–16)	16 (9.75–19.25)	<0.05
Number of harvested nodal station (median, IQR)	5 (4–6)	5 (4–5.5)	5 (4–6)	0.8

We then proceeded with the univariable analysis with the following features: gender, age, smoking status, previous cancer, tumor location, tumor diameter, PET uptake, the number of resected lymph nodes, histology, and adenocarcinoma subtype. The variables that resulted statistically significant in terms of upstaging rate were the number of resected lymph nodes (OR=2.212, 95% CI 1.074-4.555; p=0.03) and the micropapillary/solid adenocarcinoma subtype (OR=2.039, 95% CI 0.979-4.246; p=0.04, **Table 2**).

**Table 2:** Univariate analysis.

Variables	OR	p-value	95%CI
Age	1.128	0.249	0.857 – 1.341
Sex	0.975	0.187	0.625 – 1.266
Actual smokers	1.372	0.391	0.666 – 2.870
Previous cancers	1.125	0.231	0.875 – 1.178
Side	0.987	0.432	0.553 – 1.347
Tumor diameter	1.137	0.193	0.659 – 1.148
PET CT Uptake	1.012	0.102	0.898 – 1.101
Histology	1.098	0.103	0.876 – 1.231
ADC subtype	2.039	0.04	0.979 – 4.246
Number of resected nodes	2.212	0.03	1.074 – 4.555
Number of harvested nodal station	1.012	0.591	0.503 – 1.354

The multivariable analysis, summarized in **Table 3**, confirmed the predictive value of the number of resected lymph nodes (OR=2.545, 95%CI 1.136-5.701; p=0.02) and the high grade pattern of adenocarcinoma (OR=2.717, 95%CI 1.256-5.875; p=0.01).

**Table 3:** Multivariate analysis.

Variables	OR	p-value	95%CI
Histological subtypes (micropapillary and solid adc vs others)	2.717	0.01	1.256 – 5.875
Number of resected nodes (cut-off: 13, ROC analysis)	2.545	0.02	1.136 – 5.701

## 4 Discussion

Nodal upstaging in early-stage non-small cell lung cancer (NSCLC) can have a significant impact on disease management (8, 9). In this study, we aimed to analyze the clinical and histological features associated with nodal upstaging in a homogeneous cohort of patients with resected stage I NSCLC. We found that certain subtypes of adenocarcinoma, specifically the micropapillary and solid subtypes, were significantly associated with unforeseen nodal metastasis at the time of surgery. Additionally, the number of resected lymph nodes was found to be correlated with upstaging. These findings have important implications for treatment decisions and patient outcomes.

The aggressive patterns of adenocarcinoma represented by the micropapillary and solid subtypes, which were observed in at least 30% of the patients, were more likely to conceal unexpected nodal metastasis in early-stage clinical I NSCLC. These findings are consistent with previous studies that have demonstrated the prognostic role of adenocarcinoma subtypes in the early-stage setting (10). The association of specific subtypes with nodal metastasis could be relevant in guiding the decision for a more extensive resection or in assessing the need for adjuvant treatment (11). Furthermore, our analysis revealed that a higher number of resected lymph nodes was associated with upstaging. This underscores the importance of performing an adequate lymphadenectomy in improving the oncological outcomes of early-stage NSCLC patients (12). These findings align with other studies that have examined the number of harvested nodes at surgery and its association with survival (13). Patients who had a greater number of resected lymph nodes appeared to have better outcomes, and systematic lymphadenectomy with more than 10 harvested lymph nodes resulted in improved survival outcomes, particularly in a specific subgroup of patients with tumor diameter less than 20mm (14).

Despite a low rates of mediastinal N2 disease in early-stage NSCLC when following strict preoperative staging guidelines was found, the systematic mediastinal lymph node dissection for accurate staging and individualized treatment planning is of paramount importance. While imaging has improved, microscopic metastases can be missed, underscoring the need for lymphadenectomy. The inclusion of mediastinal lymph node dissection remains critical in achieving optimal outcomes for patients with early-stage NSCLC.

In our recent study concerning patients with stage I and II lung adenocarcinoma, who exhibit clinical node negativity, we have discovered intriguing connections between certain genomic features, such as ALK rearrangements, and the prediction of unexpected nodal metastasis. While these outcomes necessitate validation through a more extensive patient cohort, these revelations emphasize the significance of tumor biology (15). This importance extends beyond just medical treatment, as it aids in patient stratification even prior to surgical interventions. In our present analysis, our focus was directed at clinical stage I NSCLC including the squamous histology. We meticulously examined both histological and clinical factors that are readily accessible within routine medical practice (16). However, looking ahead, it is imperative to delve further into this specific population. Utilizing a larger patient cohort, thorough investigation into genomic biomarkers as potential prognostic indicators and predictors of upstaging should be a priority for future research endeavors.

Although our study provides valuable insights, it is essential to acknowledge its limitations. Firstly, the study design was retrospective, which inherently introduces certain limitations and potential biases. Additionally, the analysis was based on data from a single-center, which may limit the generalizability of the findings to other populations. Moreover, the sample size of the cohort was relatively small, which may limit the statistical power and precision of the results.

The study’s exclusion of patients who did not undergo systemic lymph nodes dissection (LND) raises questions regarding the potential impact of this criterion on the results. By excluding individuals who did not meet this criterion, it becomes difficult to assess the role and effectiveness of systemic LND in revealing nodal upstaging. The study lacks the necessary comparison between patients who underwent LND and those who did not, making it challenging to determine the extent to which systemic LND contributed to the identification of unexpected nodal diseases. Furthermore, the absence of perioperative and follow-up data prevents us from understanding the risk-benefit profile of LND and its potential impact on patient survival.

The primary focus of the study was on patients who underwent robotic surgery for early-stage NSCLC at our institution. This specificity limits the generalizability of the findings to other surgical approaches such as video-assisted thoracic surgery (VATS) or open surgery. The outcomes and implications of different surgical techniques may vary due to variations in procedures, instrumentation, and surgeon expertise. Therefore, caution is warranted when applying the study’s results to patients undergoing VATS or open surgery.

Lastly, given the retrospective nature of the study, another limitation of the study is that the determination of histological tumor types was frequently made post-surgery, rather than prior to the surgical procedure. This raises concerns about the practical implementation of the findings in guiding lymph node management during surgery. In order to guide lymph node dissection accurately and effectively during the operation, histological analysis of the tumor should ideally be performed before surgery. This would provide crucial information about the tumor characteristics, such as histological subtype or molecular markers, which could aid in determining the extent and approach of lymph node evaluation.

However, it is worth noting that despite these limitations, our findings align with those of previous studies that have examined early stages of NSCLC. Furthermore, efforts were made to minimize bias by including a homogeneous cohort of patients who underwent surgery according to the latest oncological guidelines.

In conclusion, our study demonstrates that the presence of the micropapillary and solid patterns of adenocarcinoma, as well as the number of resected lymph nodes, are statistically associated with the risk of unexpected nodal metastasis in a specific cohort of patients with clinical stage I NSCLC. If confirmed in larger cohorts, these findings could have implications in stratifying stage I NSCLC patients and guiding appropriate oncological treatment decisions. Moreover, the importance of performing an adequate lymphadenectomy cannot be understated, as it helps prevent inadequate staging, facilitates the appropriate administration of adjuvant treatment, and ensures optimal patient outcomes. These findings highlight the need for further research and validation in larger, multicenter studies to strengthen the evidence base and inform clinical practice in the management of early-stage NSCLC.

## Data availability statement

The raw data supporting the conclusions of this article will be made available by the authors, without undue reservation.

## Ethics statement

The studies involving human participants were reviewed and approved by Ethics committee of the IRCCS Regina Elena National Cancer Institute; Approval Code: 1465/21 Approval Date: 23 February 2021. The patients/participants provided their written informed consent to participate in this study.

## Author contributions

Conceptualization, FG, RT.; methodology, FG, EM, RT, IS; software, DF, RT, FLC; validation, FFu, FC, VA; formal analysis, FG, DM, DF, IS; investigation, FC, DM, PV; resources, FFa, EM, GA, FLC; data curation, FG, RT, VA; writing—original draft preparation, FG, RT, EM, DF; writing—review and editing, EM, FFa, FC, DF; visualization, DM, FFu, PV; supervision, FFa, GA, EM; project administration, EM, FFa; funding acquisition, FFa; All authors have read and agreed to the published version of the manuscript.
